# National Gay Men’s HIV/AIDS Awareness Day — September 27, 2017

**DOI:** 10.15585/mmwr.mm6637a1

**Published:** 2017-09-22

National Gay Men’s HIV/AIDS Awareness Day is observed each year on September 27 to direct attention to the ongoing and disproportionate impact of human immunodeficiency virus infection (HIV) and acquired immunodeficiency syndrome (AIDS) on gay, bisexual, and other men who have sex with men (MSM) (https://www.cdc.gov/hiv/risk/gender/msm) in the United States. MSM represent approximately 2% of the U.S. population (*1*); however, in 2015, MSM accounted for 69.8% of all new diagnoses including 3.0% who were also persons who inject drugs (*2*).

In 2014, among all persons living with HIV infection, an estimated 615,400 were MSM (*3*). Of these MSM, an estimated 17% had undiagnosed HIV infection. Among 358,151 MSM living with diagnosed HIV in 38 jurisdictions with complete reporting of CD4 and viral load data at year-end 2014, 58% were retained in continuous care, and 61% were virally suppressed (<200 copies of HIV RNA/mL detected at the most recent viral load test) (*3*).

CDC supports a range of measures to reduce HIV infection among MSM (https://www.cdc.gov/hiv/group/msm/index.html). Information about National Gay Men’s HIV/AIDS Awareness Day is available at https://www.cdc.gov/features/ngmhaad.

## References

[1] Purcell DW, Johnson CH, Lansky A, Estimating the population size of men who have sex with men in the United States to obtain HIV and syphilis rates. Open AIDS J 2012;6(Suppl 1:M6):98–107 . 10.2174/1874613601206010098 23049658 PMC3462414

[2] CDC. Diagnoses of HIV infection in the United States and dependent areas, 2015. HIV surveillance report, vol. 27. Atlanta, GA: US Department of Health and Human Services, CDC; 2015. https://www.cdc.gov/hiv/library/reports/hiv-surveillance.html

[3] CDC. Monitoring selected national HIV prevention and care objectives by using HIV surveillance data—United States and 6 dependent areas, 2015. HIV surveillance supplemental report, vol. 22, no. 2. Atlanta, GA: US Department of Health and Human Services, CDC; 2017. https://www.cdc.gov/hiv/library/reports/hiv-surveillance.html

